# *Lactobacillus fermentum* HFY06 reduced CCl_4_-induced hepatic damage in Kunming mice

**DOI:** 10.1039/c9ra08789c

**Published:** 2019-12-20

**Authors:** Fang Li, De-Yun Lu, Qiu Zhong, Fang Tan, Wenfeng Li, Wei Liao, Xin Zhao

**Affiliations:** Chongqing Collaborative Innovation Center for Functional Food, Chongqing University of Education Chongqing 400067 P. R. China zhaoxin@cque.edu.cn +86-23-6265-3650; Chongqing Engineering Research Center of Functional Food, Chongqing University of Education Chongqing 400067 P. R. China; Chongqing Engineering Laboratory for Research and Development of Functional Food, Chongqing University of Education Chongqing 400067 P. R. China; College of Biological and Chemical Engineering, Chongqing University of Education Chongqing 400067 P. R. China; Department of Gastroenterology, Chengdu First People's Hospital Chengdu 610041 P. R. China; Department of Public Health, Our Lady of Fatima University Valenzuela 838 Philippines; School of Life Science and Biotechnology, Yangtze Normal University Chongqing 408100 P. R. China

## Abstract

This study was conducted to investigate the preventative effect of *Lactobacillus fermentum* HFY06 on carbon tetrachloride (CCl_4_)-induced liver injury in Kunming mice. Mice were treated with HFY06, then liver damage was induced using CCl_4_. Evaluation indicators included the activities of aspartate aminotransferase (AST), triglycerides (TG), superoxide dismutase (SOD), glutathione peroxidase (GSH-Px), and malondialdehyde (MDA) in serum; cytokines levels of interleukin-6 (IL-6), tumor necrosis factor-α (TNF-α) and interferon-γ (IFN-γ) in serum; and related gene expressions of nuclear factor-κB (NF-κB), TNF-α, cyclooxygenase-2 (COX-2), copper/zinc superoxide dismutase (Cu/Zn-SOD), manganese superoxide dismutase (Mn-SOD), and catalase (CAT). Liver tissue was stained with hematoxylin and eosin for pathological analysis. Compared with the model group, HFY06 reduced the liver index, increased the serum SOD and GSH-Px activities, and reduced the AST, TG, and MDA activities in the mice. Inflammation-related IL-6, TNF-α and IFN-γ levels were also reduced after treatment with a high dose of HFY06. Pathological observation showed that CCl_4_ damaged the mouse livers, which were significantly improved after treatment with silymarin and HFY06. qPCR also confirmed that the high dose of HFY06 (10^9^ colony-forming units [CFU] per kg per day) upregulated the mRNA expression of the antioxidant genes, Cu/Zn-SOD, Mn-SOD, and CAT, in the liver tissue and downregulated the mRNA expression of the inflammatory factors, NF-κB, TNF-α and COX-2, but HFY06 was less effective than silymarin. These findings indicate that HFY06 prevented CCl_4_-induced liver damage *in vivo* but was less effective than silymarin. Thus, HFY06 may have a potential role in treating liver diseases.

## Abstract 2

This study was conducted to investigate the preventative effect of *Lactobacillus fermentum* HFY06 on carbon tetrachloride (CCl_4_)-induced liver injury in Kunming mice.

## Graphical abstract

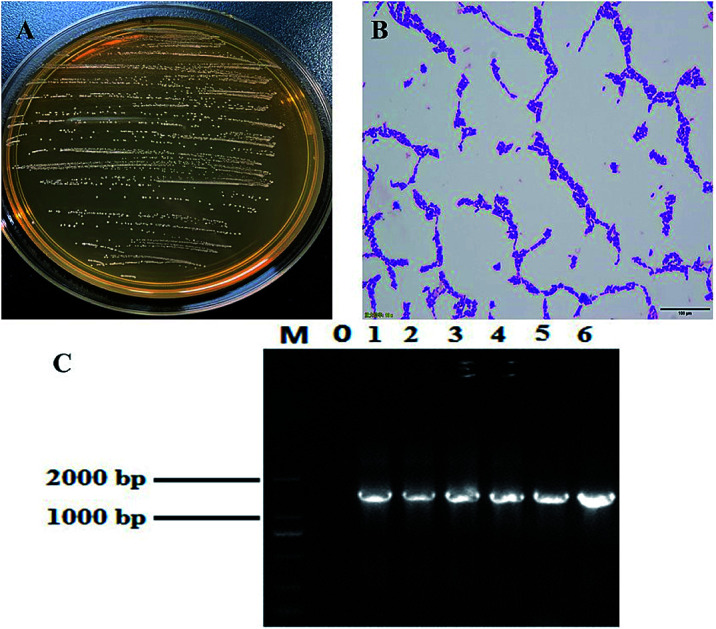

## 1. Introduction

The liver performs metabolic functions in the body. It plays important roles in maintaining human health *via* oxidation, glycogen storage, detoxification, immunity, and protein secretion.^1–3^ It is also susceptible to a range of stimuli, including viruses, toxins, drugs, alcohol and trauma, which can eventually lead to acute or chronic liver damage.^4,5^ For example, excessive use of carbon tetrachloride (CCl_4_) can cause severe liver damage, which may lead to liver failure and even death.^4,6^ Therefore, seeking methods to prevent liver damage is of great significance. Although many synthetic drugs can be used to treat liver damage, they have adverse effects on the body. Probiotics and some new biopharmaceuticals are being extensively studied as new treatments. Increasing evidence shows that probiotics benefit host health. For example, *Lactobacillus plantarum* can maintain the growth rate of infant mice during chronic malnutrition.^7^ Chen *et al.*^8^ showed that *Lactobacillus fermentum* and *Lactobacillus plantarum* protect against and attenuate CCl_4_-induced acute liver injury. Studies have shown that probiotics, such as *Lactobacillus rhamnosus*,^9^*Lactobacillus acidophilus*,^10^*Lactobacillus casei*,^11^ improve the adhesion characteristics and inflammatory responses caused by chemical drugs in liver diseases.^8,12^

Detection of aspartate aminotransferase (AST) and triglycerides (TG) in the blood is the standard method for measuring liver damage levels.^4^ During liver damage, inflammatory factors, such as interleukin-6 (IL-6), interferon-γ (IFN-γ) and tumor necrosis factor-α (TNF-α), are produced and released from numerous cells. These inflammatory factors may be concentrated around the liver and can indicate the extent of the liver damage.^13^ Increased secretion of genes such as nuclear factor-κB (NF-κB), TNF-α, cyclooxygenase-2 (COX-2) can damage and harm the liver causing inflammatory.^4,14,15^ Manganese superoxide dismutase (Mn-SOD) and copper/zinc superoxide dismutase (Cu/Zn-SOD) are SOD isomers in the body and are radical scavengers with different ions as active centers. SOD can inhibit free radicals in the body and prevent liver damage.^16,17^

*Lactobacillus fermentum* HFY06 is a lactic acid bacterium that our research team isolated and identified from natural fermented yak yogurt. In this study, we used CCl_4_ to establish a mouse model of chemical liver injury to investigate the preventive effect of *Lactobacillus fermentum* HFY06 on liver injury in mice. The results may provide insight for developing probiotic preparations. However, because a wide variety of probiotics are available, the therapeutic mechanisms and liver disease mechanisms require further research.

## 2. Materials and methods

### 2.1. Isolation and identification of *Lactobacillus*

In this experiment, yak yogurt were collected from Hongyuan, Sichuan, China. Take 1 mL of the reconstituted yogurt sample into 9 mL of sterile physiological saline, mix thoroughly, gradient dilution, take 100 μL of the dilutions of 10^−4^, 10^−5^, 10^−6^, and 10^−7^ and coat them on MRS agar plates respectively. After incubation for 24–48 hours at 37 °C, the colony morphology was observed. Select a single colony for culture. The above steps are repeated until a single colony of similar morphology was obtained. Pure colonies were inoculated with MRS (DeMan–Rogosa–Sharpe) liquid medium (5 mL) and cultured at 37 °C for 18–24 hours. Centrifuge the above 1 mL culture medium at 12 000 rpm for 1 min, discard the supernatant, add 200–500 μL sterile physiological saline, make a slice, Gram staining, microscopic examination. Moreover, the suspected purified target strain was re-inoculated into MRS liquid culture medium (5 mL). After 18–24 hours at 37 °C, DNA was extracted (Tianjian Biotechnology (Beijing) Co., Ltd., Beijing, China). The 16S rDNA gene of lactic acid strain was amplified by PCR, and the product was checked by agarose gel electrophoresis. The conditions for amplification refer to Li *et al.*^18^

### 2.2. Laboratory strain

*Lactobacillus fermentum* HFY06 is preserved in the China General Microbiological Culture Collection Center (CGMCC No. 16636), Beijing, China. *Lactobacillus delbrueckii* subspecies *bulgaricus* (LB; CGMCC no. 1.16075) was used as a comparative strain for HFY06.

### 2.3. Mouse model of hepatic damage *in vivo*

Six-week-old male Kunming mice (Laboratory Animal Center of Chongqing Medical University, Chongqing, China) of similar weights (30–35 g) were randomly divided into 6 groups of 10 mice each as follows: normal, model, silymarin (Shanghai Yuanye Bio-Technology Co., Ltd., Shanghai, China) (administered 50 mg kg^−1^ silymarin), LB (administered 10^9^ colony-forming units CFU kg^−1^*Lactobacillus delbrueckii* subspecies *bulgaricus*), HFY06-H (administered 10^9^ CFU kg^−1^*Lactobacillus fermentum* HFY06), and HFY06-L (administered 10^8^ CFU kg^−1^*Lactobacillus fermentum* HFY06).

The mice were acclimated to laboratory conditions for one week before beginning prophylactic treatment. Mice in the normal and model groups were intragastrically administered 10 mL kg^−1^ normal saline. Mice in the silymarin group received 50 mg kg^−1^ silymarin solution daily. Mice in the LB group received 10^9^ CFU kg^−1^*Lactobacillus delbrueckii* subspecies *bulgaricus*. Mice in the HFY06-H and HFY06-L groups received 10^9^ CFU kg^−1^ and 10^8^ CFU kg^−1^, respectively, of *Lactobacillus fermentum* HFY06 for two weeks. All groups except the normal group were intraperitoneally injected with CCl_4_ (10 mL kg^−1^, CCl_4_ : peanut oil at 0.8 : 100 v/v) on day 14, then all mice were fasted but allowed to drink water. After fasting for 12 h, the mice were sacrificed, serum was prepared *via* centrifugation (4 °C, 3000 rpm for 15 min), and their livers were isolated for later use.^19^ The liver organ index was the ratio of liver tissue weight to final weight of the mouse.^20,21^ The Ethics Committee of Chongqing Medical University (no. SYXK 2018-0003, Chongqing, China) approved this study.

### 2.4. Measurement of serum biochemical parameters

Serum was collected from the mice and stored at −80 °C. The serum levels of AST (C010-2-1), TG (C017-2-1), SOD (A001-1-2), GSH-Px (A005-1-2), and MDA (A003-1-2) were determined using kits (Nanjing Jiancheng Institute of Bioengineering, Jiangsu Nanjing).

### 2.5. Measurement of serum cytokine levels

Cytokine levels were assayed using IL-6 (ml002293), TNF-α (ml101826), and IFN-γ (ml22709) cytokine assay kits from Shanghai Enzyme-linked Biotechnology Co., Ltd., Shanghai, China.

### 2.6. Pathological observation of the liver

Liver samples of 1 cm^2^ thickness were removed from each mouse and immediately fixed in 10% neutral formalin fixative for 48 h, then dehydrated in 95% ethanol for 24 hours. The tissue was processed, embedded in paraffin, sectioned, and stained with hematoxylin and eosin for histopathological analysis.^22^

### 2.7. Quantitative PCR (qPCR) assay

Approximately 50 mg of liver tissue was used for the homogenate. Total RNA was isolated and extracted from the liver homogenate using TRIzol reagent per the manufacturer's instructions (Invitrogen, Carlsbad, CA, USA). The total RNA concentration was determined using a microspectrophotometer. The total RNA was used as a template and reverse transcribed to synthesize cDNA. The cDNA was stored at −80 °C and used as a template for PCR amplification. Temperature-gradient PCR was used to determine the annealing temperature for each target gene, then qPCR was performed. GAPDH was used as an internal reference, and the relative expression level of each gene was calculated using the 2^−ΔΔ*C*_t_^ method.^23^Table 1 shows the corresponding gene primer sequence.

**Table 1:** Sequences of primers used in the qPCR assay

Gene name	Sequence
Cu/Zn-SOD	Forward: 5′-AACCAGTTGTGTTGTCAGGAC-3′
	Reverse: 5′-CCACCATGTTTCTTAGAGTGAGG-3′
Mn-SOD	Forward: 5′-CAGACCTGCCTTACGACTATGG-3′
	Reverse: 5′-CTCGGTGGCGTTGAGATTGTT-3′
CAT	Forward: 5′-GGAGGCGGGAACCCAATAG-3′
	Reverse: 5′-GTGTGCCATCTCGTCAGTGAA-3′
COX-2	Forward: 5′-GGTGCCTGGTCTGATGATG-3′
	Reverse: 5′-TGCTGGTTTGGAATAGTTGCT-3′
NF-κB	Forward: 5′-ATGGCAGACGATGATCCCTAC-3′
	Reverse: 5′-CGGAATCGAAATCCCCTCTGTT-3′
TNF-α	Forward:5′-GACCCTCAGACTCAGATCATCCTTCT-3′
	Reverse: 5′-ACGCTGGCTCAGCCACTC-3′
GAPDH	Forward: 5′-AGGTCGGTGTGAACGGATTTG-3′
	Reverse: 5′-GGGGTCGTTGATGGCAACA-3′

### 2.8. Statistical analysis

The data are expressed as the mean ± SD. SPSS 22 software (IBM Corporation, North Castle, NY, USA) was used for the analysis of variance with Duncan's new multiple-range test. *P* < 0.05 was considered statistically significant. All figures were drawn using Origin 8.0 software.^24^

## 3. Results

### 3.1. Isolation and identification of HFY06

**Fig. 1:**
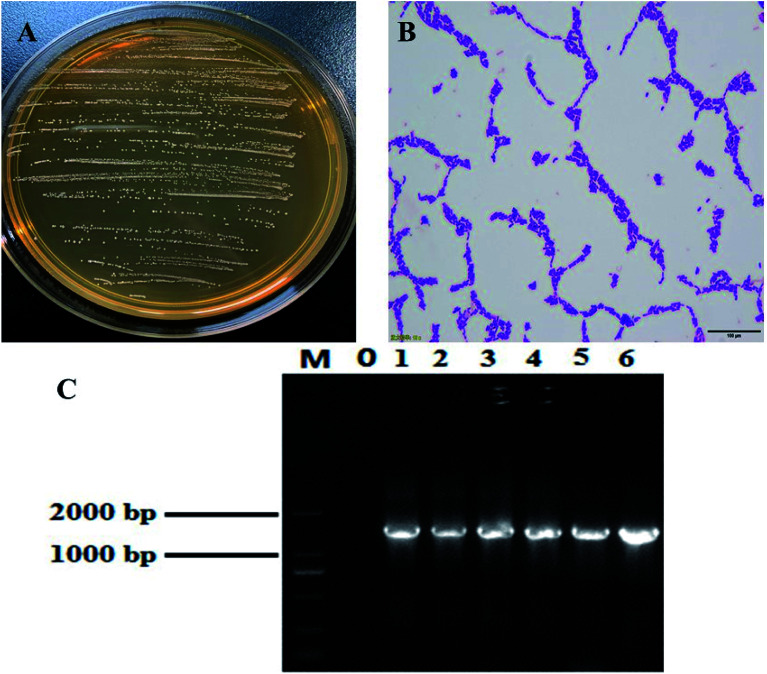
(A) Colony morphology, (B) Gram staining result, and (C) 16S rDNA agarose gel electrophoresis of PCR amplified product of *Lactobacillus fermentum* HFY06. M: 2000 bp DNA ladder; 0: negative control group; 6: *Lactobacillus fermentum* HFY06.

Most of the colonies were round white or milky white, with clean edges, moist and smooth surfaces (Fig. 1A). *Lactobacillus* was initially identified by Gram staining. Under the microscope, it mainly showed long and short rod shapes (Fig. 1B). Agarose gel electrophoresis results of 16S rDNA PCR products of this strain (Fig. 1C). A DNA fragment with the expected length of ∼1500 bp was amplified from this strain. BLAST analysis of the DNA sequencing, results showed that the strain has 99% homology with a known lactic acid bacteria listed in the GenBank database (GenBank number: NC_010610.1). The strain was named *Lactobacillus fermentum* HFY06.

### 3.2. Organ indices

The normal group had the lowest average liver weight and liver weight/body weight ratio among all groups (Table 2). After CCl_4_ treatment, the model group mice had the highest average liver weight and highest liver organ index. After treatment, the average liver weight and liver organ index of the silymarin group were closest to those of the normal group mice, followed by mice in the HFY06-H treatment group. The HFY06-L and LB treatment groups did not significantly differ, and their results were closer to those of the model group.

**Table 2:** Liver organ index of mice in each group (*n* = 10)^a^

Group	Liver weight/g	Body weight/g	Liver weight/body weight (%)
Normal	1.37 ± 0.04^a^	39.17 ± 1.39b^c^	3.49 ± 0.15^a^
Model	1.74 ± 0.04^d^	35.79 ± 1.52^a^	4.87 ± 0.11^d^
Silymarin	1.64 ± 0.14^bc^	41.03 ± 1.77^c^	3.99 ± 0.19^b^
LB	1.72 ± 0.05^d^	37.71 ± 2.07^ab^	4.61 ± 0.20^c^
HFY06-H	1.62 ± 0.03^b^	38.64 ± 1.54^b^	4.19 ± 0.20^b^
HFY06-L	1.71 ± 0.06^cd^	38.11 ± 1.14^b^	4.51 ± 0.06^c^

a Values presented are the means ± SD. ^a–d^ In the same column, values with different letters in the same column are significantly different (*p* < 0.05) and those with the same letter in the same column are not significantly different (*p* > 0.05) according to Duncan's multi-range test. Normal = normal mice; model = mice treated with CCl_4_ (0.8%); silymarin: 50 mg kg^−1^ silymarin treatment; HFY06 = mice treated with CCl_4_ (15th day) and doses (L, H) of *Lactobacillus fermentum* HFY06 (10^8^, 10^9^ CFU per kg per day); LB = mice treated with CCl_4_ (15th day) and *Lactobacillus delbrueckii* subsp. *bulgaricus* (10^9^ CFU per kg per day).

### 3.3. SOD, GSH-Px, MDA, TG and AST measurements

The normal group had the highest serum SOD and GSH-Px activities and the lowest MDA content (Table 3). The indexes in the model group were opposite those of the normal group. Compared with the model group, HFY06-H significantly increased (*P* < 0.05) the antioxidant serum SOD and GSH-Px levels and significantly decreased (*P* < 0.05) the lipid oxidation end-product MDA content, but its effect was slightly less than that of the silymarin (positive control) group. The GSH-Px and MDA levels in the HFY06-L group were similar to those of the LB group.

**Table 3:** The activities of SOD, GSH-Px and MDA in serum of mice (*n* = 10)^a^

Group	SOD (U mL^−1^)	GSH-Px (mol L^−1^)	MDA (nmol mL^−1^)
Normal	268.15 ± 7.82^f^	1662.31 ± 36.15^d^	8.31 ± 0.79^a^
Model	171.53 ± 21.22^a^	1270.71 ± 94.49^a^	18.23 ± 1.96^d^
Silymarin	251.87 ± 15.28^e^	1534.70 ± 74.00^c^	9.49 ± 1.03^a^
LB	215.43 ± 9.80^c^	1370.61 ± 79.58^ab^	15.99 ± 0.78^c^
HFY06-H	232.49 ± 3.98^d^	1439.23 ± 47.51^bc^	12.41 ± 1.51^b^
HFY06-L	199.45 ± 8.68^b^	1350.00 ± 75.41^ab^	15.15 ± 0.83^c^

a Values presented are the means ± SD. ^a–f^ In the same column, values with different letters in the same column are significantly different (*p* < 0.05) and those with the same letter in the same column are not significantly different (*p* > 0.05) according to Duncan's multi-range test. Normal = normal mice; model = mice treated with CCl_4_ (0.8%); silymarin: 50 mg kg^−1^ silymarin treatment; HFY06 = mice treated with CCl_4_ (15th day) and doses (L, H) of *Lactobacillus fermentum* HFY06 (10^8^, 10^9^ CFU per kg per day); LB = mice treated with CCl_4_ (15th day) and *Lactobacillus delbrueckii* subsp. *bulgaricus* (10^9^ CFU per kg per day).

Table 4 shows the serum AST indicators and TG levels associated with hepatoprotection. Silymarin is widely used to treat liver damage and was used as a positive control. The AST and TG were significantly increased in the model group mice (*P* < 0.05), resulting in severe liver damage. Serum levels of AST and TG were significantly lower in the silymarin and HFY06-H group mice than in the model mice but were higher than those of the normal mice. However, in all treatment groups, serum levels of AST and TG in LB were closer to the model group, indicating that the treatment effect was not very satisfactory.

**Table 4:** The levels of TG and AST in serum of mice (*n* = 10)^a^

Group	TG (nmol L^−1^)	AST (U L^−1^)
Normal	0.79 ± 0.11^a^	18.86 ± 4.16^a^
Model	1.23 ± 0.09^d^	72.52 ± 9.05^e^
Silymarin	0.81 ± 0.02^b^	58.58 ± 4.31^b^
LB	1.14 ± 0.07^c^	69.39 ± 3.77^d^
HFY06-H	0.99 ± 0.11^b^	63.91 ± 6.15^c^
HFY06-L	1.05 ± 0.18^b^	67.53 ± 3.07^d^

a Values presented are the means ± SD. ^a–d^ In the same column, values with different letters in the same column are significantly different (*p* < 0.05) and those with the same letter in the same column are not significantly different (*p* > 0.05) according to Duncan's multi-range test. Normal = normal mice; model = mice treated with CCl_4_ (0.8%); silymarin: 50 mg kg^−1^ silymarin treatment; HFY06 = mice treated with CCl_4_ (15th day) and doses (L, H) of *Lactobacillus fermentum* HFY06 (10^8^, 10^9^ CFU per kg per day); LB = mice treated with CCl_4_ (15th day) and *Lactobacillus delbrueckii* subsp. *bulgaricus* (10^9^ CFU per kg per day).

### 3.4. TNF-γ, TNF-α, and IL-6 measurements

The serum cytokine levels of IL-6, TNF-α and IFN-γ were lowest in the normal mice and highest in the model mice (Table 5). The serum IL-6, TNF-α and IFN-γ levels of mice in the silymarin and HFY06-H groups were significantly lower (*P* < 0.05) than those in the other groups and closer to those in the normal group.

**Table 5:** The levels of TNF-γ, TNF-α and IL-6 in serum of mice (*n* = 10)^a^

Group	TNF-γ (ng L^−1^)	TNF-α (ng L^−1^)	IL-6 (pg ml^−1^)
Normal	844.82 ± 84.90^a^	457.98 ± 81.35^a^	86.96 ± 12.18^a^
Model	1441.71 ± 122.83^d^	1163.64 ± 19.14^f^	145.39 ± 14.61^e^
Silymarin	902.59 ± 81.92^a^	862.99 ± 73.80^b^	105.31 ± 14.91^b^
LB	1324.79 ± 105.73^c^	956.58 ± 77.14^d^	136.42 ± 10.03^d^
HFY06-H	1090.22 ± 86.51^b^	916.77 ± 93.18^c^	107.94 ± 13.16^b^
HFY06-L	1328.80 ± 144.15^c^	1054.16 ± 95.13^e^	127.03 ± 9.43^c^

a Values presented are the means ± SD. ^a–e^ In the same column, values with different letters in the same column are significantly different (*p* < 0.05) and those with the same letter in the same column are not significantly different (*p* > 0.05) according to Duncan's multi-range test. Normal = normal mice; model = mice treated with CCl_4_ (0.8%); silymarin: 50 mg kg^−1^ silymarin treatment; HFY06 = mice treated with CCl_4_ (15th day) and doses (L, H) of *Lactobacillus fermentum* HFY06 (10^8^, 10^9^ CFU per kg per day); LB = mice treated with CCl_4_ (15th day) and *Lactobacillus delbrueckii* subsp. *bulgaricus* (10^9^ CFU per kg per day).

### 3.5. Pathological observation of the mouse liver

**Fig. 2:**
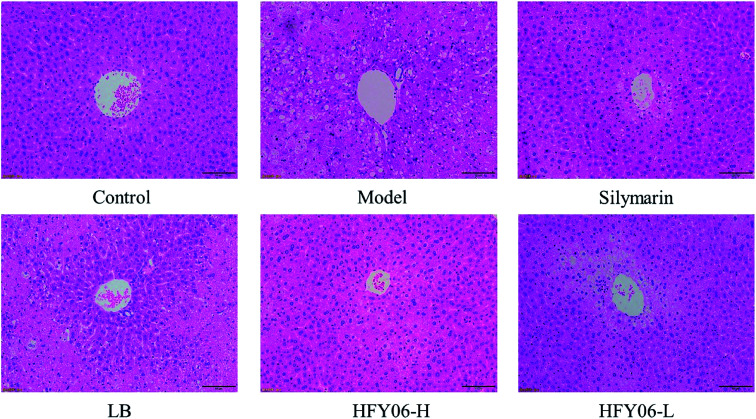
H&E pathological observation of liver in mice.

Fig. 2 shows the histological micrographs of the liver. In the normal group, the livers were normal in color, the hepatocytic cytoplasm was rich, the cell shape was regular, the size and staining were uniform, there were no signs of inflammation, the hepatocytes were arranged orderly, and the boundaries were clear. The model group hepatocytes had watery cytoplasm, with necrotizing bacteria and inflammatory cell infiltration around the central vein. In the silymarin and HFY06-H groups, the liver cells were significantly improved, and those of the silymarin group were the closest to normal. The HFY06-L group was less effective than was the HFY06-H group, and hepatocytes showed bleeding in the area around the lobular vein.

### 3.6. Gene expressions of Cu/Zn-SOD, Mn-SOD and CAT in the mouse livers

**Fig. 3:**
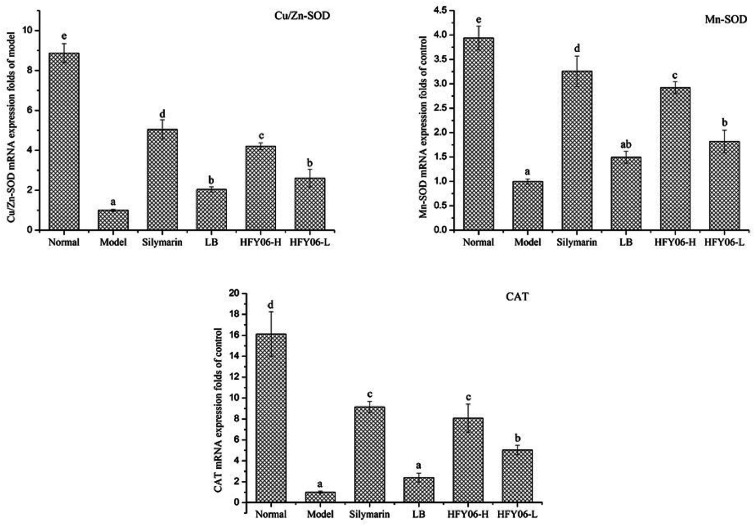
mRNA expression level of Cu/Zn-SOD, Mn-SOD and CAT in mouse liver.

qPCR showed that CCl_4_ reduced the mRNA expressions of Cu/Zn-SOD, Mn-SOD and CAT (Fig. 3). Cu/Zn-SOD, Mn-SOD and CAT expressions were lower in the model group than in the normal group. After silymarin, LB and HFY06 treatment, the mRNA expressions of Cu/Zn-SOD, Mn-SOD and CAT increased, and those of the silymarin group were near those of the normal mice, followed by the high-dose (HFY06-H), low-dose (HFY06-L) and LB treatment groups.

### 3.7. Gene expressions of COX-2, NF-κB, and TNF-α in the mouse livers

**Fig. 4:**
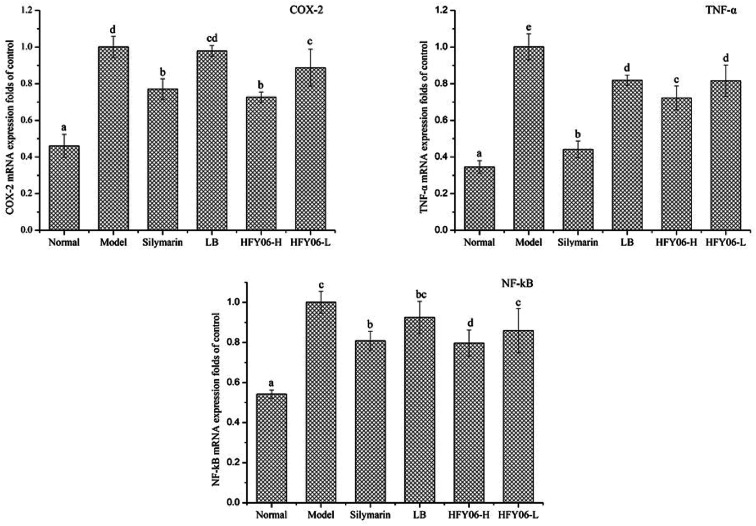
mRNA expression level of COX-2, NF-κB, and TNF-α in mouse liver.

Fig. 4 shows the gene expressions of COX-2, TNF-α and NF-κB in the mouse liver tissue. The expression levels of COX-2, TNF-α and NF-κB in the model group were significantly higher than those of the normal group (*P* < 0.05). Silymarin, LB and HFY06 significantly regulated the expressions of COX-2, TNF-α and NF-κB inflammation-related genes compared with those of the model group (*P* < 0.05). These results suggest that silymarin and HFY06 can prevent liver injury by enhancing anti-inflammatory activity. The effect of HFY06-H was better than that of HFY06-L at the same dose.

## 4. Discussion

Lactic acid bacteria (LAB) exert special physiological functions by producing organic acids, special enzymes, and other substances.^25^ LAB are widely used in food, medicine, bioengineering, agriculture, industry, and health products.^26,27^ Probiotics are reported to protect against chronic liver damage caused by alcohol, viral infections and metabolic diseases.^28,29^ Recent studies have shown that consuming LAB, such as bifidobacteria, can alleviate lipopolysaccharide and d-galactose-induced acute liver injury.^30,31^ Another study showed that combining *Lactobacillus plantarum* and l-arginine can protect against endotoxin-induced liver damage.^32^ CCl_4_-induced liver injury can cause significant intrahepatic inflammation and liver fibrosis in mice.^4,15^ Therefore, we used a CCl_4_-induced oxidative stress and acute liver injury mouse model to explore the protective effect of *Lactobacillus fermentum* HFY06 isolated from yak yogurt on liver injury.

Liver weight and liver indices were used as indicators of CCl_4_-induced liver injury.^14,33^ The results showed that a high dose of *Lactobacillus fermentum* HFY06 reduced the liver weight and liver indices in mice with CCl_4_-induced liver injury. These effects were similar to those of silymarin, making the indices of the treatment group closer to those of the normal group. In addition, histopathology is an important clinical standard for diagnosing liver injury.^34^ We analyzed mouse liver tissue sections to effectively study and evaluate the liver-protective activity of HFY06 against CCl_4_-induced liver injury, and found that compared with the treatment effect of the LB group, HFY06 was more effective in preventing CCl4-induced liver injury.

CCl_4_ leads to trichloromethyl free radical production in liver oxidative metabolism, which attacks lipid cell membranes and damages hepatocytes *via* lipid peroxidation.^15^ Inhibiting liver tissue oxidation and reducing free radicals in liver tissue can effectively protect against tissue damage,^14^ including regulation of SOD, CAT and GSH-Px oxidation.^35^ SOD has specialized physiological activity and is the main enzyme for scavenging free radicals.^35,36^ The main biological function of GSH-Px is to reduce lipid hydroperoxides and catalyze the decomposition of hydrogen peroxide to inhibit its direct destruction of the biomembrane and alleviate cell damage.^37^ MDA is the metabolic end-product of lipid peroxidation. Therefore, the MDA index is usually used to indicate the degree of lipid peroxidation and cell damage *in vivo*.^38^ MDA content is high after liver injury *in vivo*. High-dose HFY06 significantly regulated liver injury-induced SOD, GSH-Px and MDA levels in the body to protect the liver from the effects of CCl_4_, the effect was slightly less than that of silymarin, but the effect was better than that of LB, preliminarily indicating that HFY06 have an inhibitory effect on liver injury.

AST is a transaminase marker for detecting liver injury.^2,14^ After liver cells are damaged, enzymes such as AST are released into the blood. Therefore, detection of AST activity in the serum can accurately reflect the degree of liver damage.^14^ TG levels in the liver tissue and serum also increase significantly after liver tissue damage.^4,35^ The liver functions of mice in the silymarin, LB and HFY06 treatment groups were significantly enhanced compared with those of the model group, and the AST and TG levels in the blood were significantly reduced (*P* < 0.05; Table 4). The AST index of the HFY06-H group was lower than those of the HFY06-L group, suggesting that high-dose HFY06 can effectively regulate the AST level in mice and reduce the influence of CCl_4_ on the body. These data are consistent with those of previous studies and confirm that probiotics can be used to alleviate liver injury.^39,40^

Studies have shown that after some exogenous stimuli, such as with CCl_4_-induced liver injury, monocytes produce a variety of inflammatory cytokines. Therefore, the levels of serum cytokines, including IL-6, TNF-γ and TNF-α, in patients with inflammatory diseases are higher than those in healthy individuals.^4,15^ Inflammatory factors play important roles in liver function, and reducing these inflammatory cytokines may be an improved method of preventing liver injury.^41^ Previous studies have shown that probiotics can improve alcohol-induced liver injury through their own anti-inflammatory properties.^42^ Selenium-enriched *Lactobacillus* can reduce serum TNF- α levels in rats with liver injury.^43^ In this study, silymarin, LB and HFY06 reduced some inflammation-related factors, including IL-6, TNF-γ and TNF-α. Among them, silymarin had the most obvious effect, followed by high-dose HFY06, suggesting that HFY06 can help prevent liver injury at sufficient doses.

Genes related to antioxidation in the tissues, such as Cu/Zn-SOD, Mn-SOD and CAT, can be used as gene indicators to monitor CCl_4_-induced liver oxidative damage. Studies have shown that CC1_4_-induced liver injury can be regulated or even restored to normal levels by the scavenging free radical enzyme, SOD.^2,4^ Therefore, the antioxidant effect of HFY06 can be determined by determining the levels these important antioxidant-related genes. Compared with the model group, high-dose HFY06 upregulated Cu/Zn-SOD, Mn-SOD, and CAT mRNA expression, indicating that the free radicals produced by CCl_4_-induced liver oxidative metabolism can be eliminated. Expressions of these antioxidant-related genes were near those of the silymarin treatment group, which is consistent with previous research results.^14^

NF-κB, TNF-α and COX-2 genes in tissues can be used as biomarkers to monitor visceral injury. NF-κB is a transcription factor widely existing in various cells, regulating the expression of genes related to inflammatory response and antiapoptosis.^44^ NF-κB maintains important physiological functions *in vivo* and exists in the cytoplasm where it binds to inhibitor protein IκB. After being induced by various drugs, NF-κB can be activated and translocated into the nucleus, promoting inflammation and leading to the release of TNF-α and other mediators, thus causing liver cell damage.^45^ COX-2 plays an important role in inducing inflammation. NF-κB can promote COX-2 gene transcription and regulate its expression, thereby amplifying the inflammatory response and aggravating liver injury.^14,15^ Liver inflammatory cells can produce superoxide, trigger oxidative stress, produce many reactive oxygen species, and damage cells, all of which can be regulated by several antioxidant-related genes. NF-κB, TNF-α and COX-2 mRNA expressions in the model group were higher than those in the normal group. Treatment with *Lactobacillus fermentum* HFY06 downregulated the expression of these genes to reduce damage due to inflammatory response in the liver tissue. The results of this study revealed that sufficient doses of HFY06 can prevent liver injury and inflammation and better than the commonly used LB.

## 5. Conclusion

The preventive effect of *Lactobacillus fermentum* HFY06 on liver injury can be evaluated by a CCl_4_-induced liver injury model, including determination of liver index; serum AST, GT, IL-6, TNF-α, and TNF-γ, and other indicators; pathological observation; expression of antioxidative genes such as Cu/Zn-SOD and Mn-SOD; and other inflammation-related genes such as NF-κB, TNF-α and COX-2. The effect of HFY06 was positively correlated with bacterial dose, and its effect on prevent CCl_4_-induced liver injury was better than that of LB. The results of this study can guide the application of *Lactobacillus fermentum* HFY06.

## Conflicts of interest

No conflicts of interest in this article.

## Supplementary Material
